# Supplementary material for: Cell-type specialization is encoded by specific chromatin topologies
Source: Nature. 2021 Nov 17;599(7886):684–91. doi: 10.1038/s41586-021-04081-2 (PMC8612935; doi:10.1038/s41586-021-04081-2)
Supplement: Supplementary file 1 — Reporting Summary [file 41586_2021_4081_MOESM1_ESM.pdf]

nature research

Corresponding author(s): Ana Pombo, Warren Winick-Ng

Last updated by author(s): Aug 27, 2020

Reporting Summary

Nature Research wishes to improve the reproducibility of the work that we publish. This form provides structure for consistency and transparency in reporting. For further information on Nature Research policies, see our Editorial Policies and the Editorial Policy Checklist.

Statistics

For all statistical analyses, confirm that the following items are present in the figure legend, table legend, main text, or Methods section.

n/a	Confirmed	
☐	☒	The exact sample size (*n*) for each experimental group/condition, given as a discrete number and unit of measurement
☐	☒	A statement on whether measurements were taken from distinct samples or whether the same sample was measured repeatedly
☐	☒	The statistical test(s) used AND whether they are one- or two-sided<br>*Only common tests should be described solely by name; describe more complex techniques in the Methods section.*
☒	☐	A description of all covariates tested
☐	☒	A description of any assumptions or corrections, such as tests of normality and adjustment for multiple comparisons
☐	☒	A full description of the statistical parameters including central tendency (e.g. means) or other basic estimates (e.g. regression coefficient) AND variation (e.g. standard deviation) or associated estimates of uncertainty (e.g. confidence intervals)
☐	☒	For null hypothesis testing, the test statistic (e.g. F, t, r) with confidence intervals, effect sizes, degrees of freedom and P value noted<br>*Give P values as exact values whenever suitable.*
☒	☐	For Bayesian analysis, information on the choice of priors and Markov chain Monte Carlo settings
☐	☒	For hierarchical and complex designs, identification of the appropriate level for tests and full reporting of outcomes
☐	☒	Estimates of effect sizes (e.g. Cohen's d, Pearson's r), indicating how they were calculated

Our web collection on statistics for biologists contains articles on many of the points above.

Software and code

Policy information about availability of computer code

Data collection	Leica Laser Microdissection v8.2; Leica Application Suite X v3.5.5.19976; BD FACSDiva v8.0.2
Data analysis	bedtools v2.29.2; UpSetR v1.4.0; Seurat v3.1.4; samtools v1.3.1; deeptools v3.1.3; DESeq2 v1.24.0; bowtie2 v2.3.4.3; 10x Genomic Cellranger v1.2.0; ArchR v0.9.1; GenomicAlignments v1.20.1; Regulatory Genomics Toolbox v0.12.3; leiden' package v0.3.3; STAR v2.4.2a, RSEM v1.2.25, Ea-utils v1.1.2-537, Picard-tools v2.5.0; Sambamba v0.6.8; GOElite v1.2.4; LAMMPS, v.5june2019; Anaconda package v.4.7.12; POV Ray v.3; Fiji software v2.0.0-rc-69/1.52p; Adobe Photoshop CS6; UCSC utilities http://hgdownload.soe.ucsc.edu/admin/exe/; MELTRON and trans-cis contact ratio pipelines were deposited in https://github.com/pombo-lab/Meltron; custom python and R scripts for GAM window calling, GAM quality control, GAM genome sampling quality and resolution, production of NPMI matrices, aggregated maps, k-means clustering, calculation of insulation score and compartment calling were deposited in https://github.com/pombo-lab/WinickNg_Kukalev_Harabula_Nature_2021

For manuscripts utilizing custom algorithms or software that are central to the research but not yet described in published literature, software must be made available to editors and reviewers. We strongly encourage code deposition in a community repository (e.g. GitHub). See the Nature Research guidelines for submitting code & software for further information.

Data

Policy information about availability of data

All manuscripts must include a data availability statement. This statement should provide the following information, where applicable:

- Accession codes, unique identifiers, or web links for publicly available datasets
- A list of figures that have associated raw data
- A description of any restrictions on data availability

Raw fastq sequencing files for all samples from DN, PGN and OLG GAM datasets, together with non-normalized co-segregation matrices, normalized pair-wise chromatin contacts maps and raw GAM segregation tables are available from the GEO repository under accession number GSE94364. Raw fastq sequencing files for

AP

mESC GAM datasets are available from 4DN data portal (https://data.4dnucleome.org/). The 4DN sample IDs for all samples used in the study are available in Supplemental Table 1.
Raw confocal and laser microdissection images, as well as images and ROIs for cryo-FISH experiments are available at: https://github.com/pombo-lab/WinickNg_Kukalev_Harabula_Nature_2021/tree/main/microscopy_images/.
Raw single cell mESC transcriptome data are available from ENA data portal (https://www.ebi.ac.uk/ena/browser/home). The ENA sample IDs for all samples used in the study are available in Supplemental Table 13. Raw single cell and bulk ATAC-seq bam-files for DNs and mESCs, respectively, are available from the GEO repository under accession number GSE174024, together with processed bigwig files. A public UCSC session with all data produced, as well as all published data utilized in this study is available at: http://genome-euro.ucsc.edu/s/Kjmorris/Winick_Ng_2021_GAMbrainpublicsession.
UCSC Table browser: http://genome.ucsc.edu/cgi-bin/hgTables; ERCCs: https://www.thermofisher.com/order/catalog/product/4456739;
Cell ranger refdata-cellranger-atac-mm10-1.2.0l: https://support.10xgenomics.com/single-cell-atac/software/pipelines/latest/advanced/references#overview;
HOCOMOCO database v11: https://hocomoco11.autosome.ru/

Field-specific reporting

Please select the one below that is the best fit for your research. If you are not sure, read the appropriate sections before making your selection.

☒ Life sciences ☐ Behavioural & social sciences ☐ Ecological, evolutionary & environmental sciences

For a reference copy of the document with all sections, see nature.com/documents/nr-reporting-summary-flat.pdf

Life sciences study design

All studies must disclose on these points even when the disclosure is negative.

Sample size

The appropriate number of samples for a GAM dataset varies and depends on multiple parameters such as nuclear volume, level of chromatin compaction, quality of DNA extraction, etc. Since most of these parameters can be assessed only after the data has been collected and processed, we recommend that the optimal resolution is defined during the collection of each GAM dataset, rather than trying to estimate optimal sample size before data collection. GAM data can be collected in multiple batches from the same starting material, therefore the sample size can be increased until the desired resolution is achieved.
Resolution is determined by comparing the distribution of intra-chromosomal co-segregation frequencies for all possible pairs of loci at a given resolution, using the standard Poisson distribution. In case multiple datasets from different samples are analyzed together, we recommend choosing the highest possible resolution appropriate for every dataset involved in the analysis. In the present study, we measured co-segregation frequencies for all GAM datasets, finding that 98.8 – 99.9% of all mappable pairs of windows were sampled at least once at 50 kb resolution considering all genomic distances. The script to test the quality of genome sampling at given resolution was uploaded to GitHub (https://github.com/pombo-lab/WinickNg_Kukalev_Harabula_Nature_2021/blob/main/code/GAM.define.working.resolution.py).
For single cell ATAC-seq data, no statistical method was used to predetermine sample size, as in https://www.nature.com/articles/s41593-018-0079-3?proof=t
For scRNA-seq (mESCs), no statistical method was used to predetermine sample size. Libraries were generated twice, from mESCs from different biological replicates, to account for experimental variability.

Data exclusions

The quality of individual GAM libraries was determined using a combination of several quality metrics: clustering of positive windows, sequencing depth and lack of sample contamination. Due to the nature of genome sampling by ultrathin cryosectioning, good quality positive windows are expected to cluster next to each other, while noise is expected to behave randomly and not cluster on the linear genome sequence. Positive windows in low quality GAM samples (i.e. from the water controls, or samples not amplified during the whole-genome amplification reaction) often do not cluster with other positive windows, termed "orphan windows". In this study, an individual GAM sample was considered to be of good quality if it had < 70% orphan windows, > 50,000 uniquely mapped reads and no sign of cross-well contamination, as determined by low Jaccard index score to the distribution of positive windows in all samples processed at the same time.
For single-cell ATACseq (midbrain VTA), single cells were considered of low quality (and removed from the analysis) if TSS enrichment score was < 4 and there were < 2500 unique fragments per cell. After processing of raw data and clustering, the DN population was identified (see Methods) and single-cell IDs were extracted. ATAC-seq fragments derived from DN single-cells were subset from the original VTA position sorted BAM file and grouped into a subset containing only DN fragments. The subset file was uploaded to GEO (GSE174024).
For scRNA-seq (mESCs), libraries were excluded from the analysis if they were derived from cells that appeared as debris or doublets/multiplets upon visual inspections of the C1 chip, or if the libraries appeared as outliers in number of sequencing reads or mapping statistics, as fully detailed in the Methods section.

Replication

For the two neuronal cell types, single animal replicates were produced and had similar results in all metrics tested. Variations in replicates are reported through the main and supplemental data.

Randomization

Randomization was not relevant to our study.The experiments and the subsequent analysis were performed on wild type animals or cell lines, where no treatment or disease comparison was performed. As described in the Methods section, our samples were processed in different labs by different people. There was no selection criteria for the wild type mice used in the study.

Blinding

Blinding was not relevant to our study. We did not perform clinical trials, nor compared disease models or different treatments. As described in the Methods section our samples were processed in different labs by different people. There was no selection criteria for the wild type mice used in the study.

Reporting for specific materials, systems and methods

AP

We require information from authors about some types of materials, experimental systems and methods used in many studies. Here, indicate whether each material, system or method listed is relevant to your study. If you are not sure if a list item applies to your research, read the appropriate section before selecting a response.

Materials & experimental systems

n/a	Involved in the study	
☐	☒	Antibodies
☐	☒	Eukaryotic cell lines
☒	☐	Palaeontology and archaeology
☐	☒	Animals and other organisms
☒	☐	Human research participants
☒	☐	Clinical data
☒	☐	Dual use research of concern

Methods

n/a	Involved in the study	
☒	☐	ChIP-seq
☐	☒	Flow cytometry
☒	☐	MRI-based neuroimaging

Antibodies

Antibodies used

Pel-Freez Arkansas, Catalog number P60101-0; Sheep anti-tyrosine Hydroxylase, Lot number ajo1217p;
Merck, Catalog number MAB3422, Mouse anti-pan-histone, clone H11-4, Lot number 2842169;
Abcam, Catalog number ab13970 Chicken anti-GFP; Lot number GR236651-21;
Invitrogen, Donkey anti-sheep Ig, AlexaFluor-488, Catalog number A-11015;
mouse anti-nucleophosmin B23 was a kind gift from Harris Busch;
Abcam, Goat anti-chicken If, AlexaFluor-488, Catalog number ab150169;
Invitrogen, Donkey anti-mouse Ig, AlexaFluor-488, Catalog number A-10035;
Invitrogen, Donkey anti-mouse Ig, AlexaFluor-555, Catalog number A-32773;

Validation

Pel-Freez Arkansas, Catalog number P60101-0, Sheep anti-tyrosine Hydroxylase, Lot number ajo1217p - validated by Western blot in rat caudate lysate (https://www.pelfreez-bio.com/wp-content/uploads/2014/07/74041-PDS-P60101-Tyrosine-Hydroxylase-Antibody-Sheep-Rev-02.pdf)
Merck, Catalog number MAB3422, Mouse anti-pan-histone, clone H11-4, Lot number 2842169 - validated by Western blot on Jurkat lysates (https://www.merckmillipore.com/DE/de/product/Anti-Histone-Antibody-clone-H11-4,MM_NF-MAB3422?ReferrerURL=https%3A%2F%2Fwww.google.com%2F&bd=1#anchor_Product%20Information)
Abcam, Catalog number ab13970, Chicken anti-GFP; Lot number GR236651-21 - validated by Western blot in whole cell lysates of mouse cardiomyocytes overexpressing a GFP plasmid (https://www.abcam.com/GFP-antibody-ab13970.html?gclsrc=aw.ds|aw.ds&gclid=CjwKCAjwmeiIBhA6EiwA-uaeFVTDLhxCilj2b-NEfOFbj1nn-BBuPJCXCP4jcjC7mY5NkInrgp6TFBoCekUQAvD_BwE)
mouse anti-nucleophosmin B23 was validated by western blot in HeLa nuclear extract (Valdez, B.C., et al. Identification of the Nuclear and Nucleolar Localization Signals of the Protein p120. J. Biol. Chem. 269, 23776-23783 (1994))

Eukaryotic cell lines

Policy information about cell lines

Cell line source(s)

The mouse embryonic stem cells clone 46C derived from E14tg2a cells were provided by Dr. Domingos Henrique from Instituto de Medicina Molecular, Faculdade Medicina Lisboa, Lisbon, Portugal

Authentication

46C E14tg2 mESCs are not listed in the ICLAC Register of Misidentified Cell Lines. The 46C E14tg2 mESC line was generated by insertion of an eGFP cassette under the control of the Sox1-promoter in E14 tg2 cells. Reads aligned with GFP sequence were identified in the GAM sequencing data from mESCs. Additionally, genome sequencing data from GAM mESC samples was mined for SNPs. Though GAM sequencing reads are sparsely distributed across the genome, there was a 64% overlap of GAM mESC SNPs with SNPs identified from the parental E14tg2 genome sequencing data (https://www.ncbi.nlm.nih.gov/sra?term=SRX389523).

Mycoplasma contamination

The cells were negative for Mycoplasma contamination. The Mycoplasma test was performed according to the manufacturer's instructions (AppliChem Cat#A3744,0020)

Commonly misidentified lines
(See ICLAC register)

No commonly misidentified cell lines were used in the study (46C E14tg2 mESC are not listed in the ICLAC Register of misidentified cell lines https://iclac.org/databases/cross-contaminations/)

Animals and other organisms

Policy information about studies involving animals; ARRIVE guidelines recommended for reporting animal research

Laboratory animals

All animals used in this study were from the species Mus musculus.
The following mouse strains were used:
- C57Bl/6NI (RRID: IMSR_CR:027; WT) for snATAC-seq experiments mice, adult male, ages 7 and 9 weeks;
C57BL/6NI mice were housed in a temperature controlled room at 22±2°C with humidity of 55±10% in individually ventilated cages with 12-hours light/12-hours dark cycles with free access to food and water ad libitum.
- C57Bl/6NCrl (RRID: IMSR_CR:027; WT) for GAM experiments, mice purchased from Charles River, adult male, 2-3 months old;
- TH-GFP mice (B6.Cg-Tg(TH-GFP)21-31/C57B6), adult male, 2-3 months old;
C57Bl/6NCrl and TH-GFP mice had access to food and water ad libitum and were kept on a 12 h:12 h day/night cycle at 20-23°C at

AP

45% (+/-5%) humidity.
- Sox10::Cre-RCE::loxP-EGFP animals were obtained by crossing Sox10::Cre animals on a C57BL/6j genetic background with RCE::loxP-EGFP animals on a C57BL/6xCD1 mixed genetic background, both mouse lines available from The Jackson Laboratories. Adult male, sacrificed at P21. The mice received regular chew diet (either R70 diet or R34, Lantmännen Lantbruk, Sweden, or CRM-P, 801722, Special Diet Services). General housing parameters such as relative humidity, temperature, and ventilation follow the European convention for the protection of vertebrate animals used for experimental and other scientific purposes treaty ETS 123. Briefly, consistent relative air humidity of 50%, 22 °C and the air quality is controlled with the use of stand-alone air handling units supplemented with HEPA filtrated air. Monitoring of husbandry parameters is done using ScanClime (Scanbur) units. Water was provided by using a water bottle, which was changed weekly.
- Satb2flox/flox mice that carry the floxed exon 4 of the Satb2 gene have been generated by microinjection of embryonic stem cells clone Satb2_G07, JM8.N4 subline from KOMP repository, into blastocysts from C57Bl/6NCrl (RRID: IMSR_CR:027; WT) mice, purchased from Charles River. Adult males, sacrificed at 19 weeks old. All mice had access to food and water ad libitum and were kept on a 12 h:12 h day/night cycle at 22.5 °C (+/-1°C) at 55% (+/-10%) humidity.

Wild animals	No wild animals were used in the study
Field-collected samples	No field-collected samples were used in the study
Ethics oversight	Experimental procedures involving C57Bl/6NI were approved by the regulations of local animal care committee (Landesamt für Gesundheit und Soziales, Berlin, Germany) and follow the Directive 2010/63/EU of the European Parliament on the protection of animals used for scientific purposes, organ preparation was done under license X9014/11. Experimental procedures involving C57Bl/6NCrl and TH-GFP animals were approved by the Imperial College London's Animal Welfare and Ethical Review Body. Experimental procedures involving Sox10::Cre-RCE::loxP-EGFP animals were performed following the European directive 2010/63/EU, local Swedish directive L150/SJVFS/2019:9, Saknr L150 and Karolinska Institutet complementary guidelines for procurement and use of laboratory animals, Dnr 1937/03-640. The procedures described were approved by the local committee for ethical experiments on laboratory animals in Sweden (Stockholms Norra Djurförsöksetiska nämnd), lic.nr. 130/15. Experimental procedures involving Satb2flox/flox mice were done according to the Austrian Animal Experimentation Ethics Board (Bundesministerium für Wissenschaft und Verkehr, Kommission für Tierversuchsangelegenheiten)

Note that full information on the approval of the study protocol must also be provided in the manuscript.

Flow Cytometry

Plots

Confirm that:

☒ The axis labels state the marker and fluorochrome used (e.g. CD4-FITC).

☒ The axis scales are clearly visible. Include numbers along axes only for bottom left plot of group (a 'group' is an analysis of identical markers).

☒ All plots are contour plots with outliers or pseudocolor plots.

☒ A numerical value for number of cells or percentage (with statistics) is provided.

Methodology

Sample preparation	Male C57Bl/6NI (RRID: IMSR_CR:027; WT) mice, ages 7 and 9 weeks, were sacrificed by cervical dislocation. Brains were removed and the tissue containing the midbrain VTA was dissected from each hemisphere at room temperature and rapidly frozen on dry ice. Frozen tissue was homogenized in 500 microL 0.1X lysis buffer (10 mM Tris-HCl, pH 7.4, 10 mM NaCl, 3 mM MgCl2, 1% BSA, 0.01% Tween-20, 0.01% Nonidet P40 Substitute 0.001% Digitonin). Chilled wash buffer (500microL, 10 mM Tris-HCl, pH 7.4, 10 mM NaCl, 2 mM MgCl2, 1% BSA, 0.1% Tween-20) was added to the lysed cells, and the suspension was passed through 30 micrometer CellTrics strainers (Th Geyer, cat# 7648779). The final ~500 microL nuclei suspension was stained with DAPI (final concentration 0.03 microg/mL) for ~5 min.
Instrument	BD FACSAria III Flow Cytometer
Software	BD FACSDiva v 8.0.2
Cell population abundance	Target population (intact nuclei) abundance was between 1-5% (see Extended Data Figure 4h-i)
Gating strategy	A first gate excluded debris in a FSC/SSC-plot and a consecutive, second gate in a DAPI-A/DAPI-H-plot was used to exclude doublets and nuclei with incomplete DNA content.

☒ Tick this box to confirm that a figure exemplifying the gating strategy is provided in the Supplementary Information.

31.8.2021 [illegible]
