# Supplementary figures and images for: Cell-type specialization is encoded by specific chromatin topologies
Source: Nature. 2021 Nov 17;599(7886):684–91. doi: 10.1038/s41586-021-04081-2 (PMC8612935; doi:10.1038/s41586-021-04081-2)

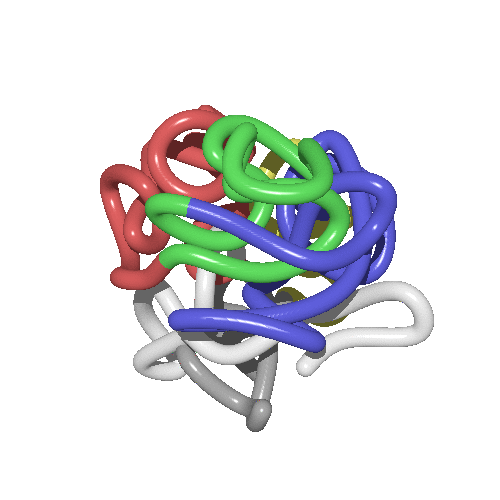

Supplement: Supplementary file 3 — Supplementary Video 1 Nrxn3 locus PRISMR model mES cells. Example polymer model for mES cells in the Nrxn3 locus (chr12:87,600,000–92,400,000). [file 41586_2021_4081_MOESM3_ESM.gif]

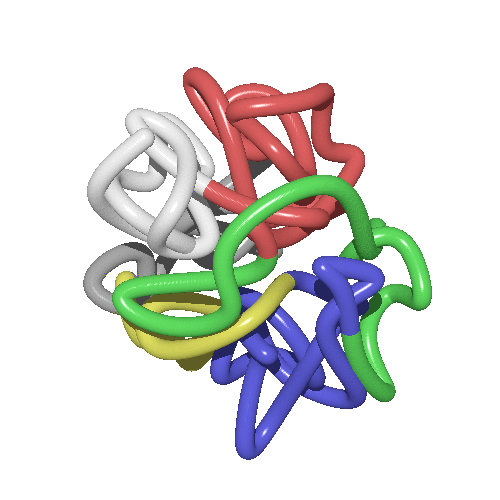

Supplement: Supplementary file 4 — Supplementary Video 2 Nrxn3 locus PRISMR model DNs. Example polymer model for DNs in the Nrxn3 locus (chr12:87,600,000–92,400,000). [file 41586_2021_4081_MOESM4_ESM.gif]
